# Job related stress among nurses working in Jimma Zone public hospitals, South West Ethiopia: a cross sectional study

**DOI:** 10.1186/s12912-016-0158-2

**Published:** 2016-06-16

**Authors:** Tadesse Dagget, Ashagre Molla, Tefera Belachew

**Affiliations:** 1 School of Nursing, Bahir Dar University, PO Box 79, Bahir Dar, Ethiopia; 2 School of Graduate Studies, Jimma University, Jimma, Ethiopia

**Keywords:** Job related stress, Nurses’ stress, Level of stress

## Abstract

**Background:**

Occupational stress exists in every profession, nevertheless, the nursing profession appears to experience more stress at work compared to other health care workers. Unmanaged stress leads to high levels of employee dissatisfaction, illness, absenteeism, high turnover, and decreased productivity that compromise provision of quality service to clients. However, there is a scarcity of information about nurses’ job stress in Jimma zone public hospital nurses.

The aim of the present study was to assess job related stress and its predictors among nurses working in Jimma Zone public hospitals, South-West Ethiopia in 2014*.*

**Method:**

An institution based cross sectional study was conducted from March 10 to April 10, 2014 through a census of nurses who are working in Jimma Zone public hospitals using a structured self-administered questionnaire. SPSS Statistics Version 20 used. For the outcome variable: overall job related stress, the participant’s responses on each item score summed: a stress score ranging from a minimum of 26 and maximum score of 116. The higher the sum the more the stressed the nurse. The level of stress calculated through tertial the lower to low stress, the middle to moderate & the higher to high stress. Moreover, bivariate and multivariable linear regressions done to see the association between the predictor (sex, age, mutual understanding at work, Job satisfaction and working unit/department) and the outcome variable (Job related stress).

**Results:**

A total of 341 nurses working in Jimma Zone public hospitals were given the questionnaire, and the response rate was 92.3 % (315). This study indicated an average overall job related stress level of 58.46 ± 12.62. The highest level of job related stress was on the sub scale of dealing with death & dying mean score of 62.94 % followed by uncertainty regarding patient treatment 57.72 % and workload 57.6 %. While job related stress from sexual harassment had the lowest mean score of 46.19 %.

**Conclusion:**

Overall job related stress varies across working unit. Working in a chronic illness follow up clinic, the mutual understanding at work between nurse & physician and job satisfaction were negatively associated predictors of job related stress.

## Background

Nurses play a pivotal role in any health care institution and encompass the largest workforce in any health care institution; they act as direct caregivers who serve a hospital twenty-four hours a day, seven days a week. This gives nurses a unique perspective on both patient care and hospital operations [1]. Nurses occupy particularly interesting position in the provision of health care. Often they are the sole intermediary between the physician and the patient and in the front line of health services [2]. Nurses work long hours; 12-h shifts are common, especially in hospitals, and the job is physically taxing. In addition, nurses deal with human suffering daily. Nursing requires a high level of vigilance to assure patient safety in an environment that is complex and may even be chaotic. Medical emergencies added to the tension of patient care, and nurses deal with grief and loss when a patient dies. All of these factors can increase a nurse’s stress level and affect nurse health [3].

Stress is often described as a feeling of being overloaded, wound-up tight, tense and worried [4]. It is a disruptive condition that occurs in response to adverse influences from the internal or external environments [5]. Stress can be experienced from four basic sources; environment, social stressors, physiological, and thoughts [6].

The working environment is one of the most important resources of occupational stress [7]. Stress at work is one of the major psychosocial risks at work. Work-related stress is a problem and is of great concern to employees, employers, psychologists and counselors [8].

Nursing, by virtue of its nature, is a profession subjected to a high degree of stress [9]. Occupational stress exists in all professions, but the nursing profession appears to experience more stress at work compared to other health-care workers [7, 10, 11]. Perceived stressful work increases the desire to leave the employer [12]. Job-stress in the nursing profession has been a global problem with rates of 9.20–68.0 % of nurses suffering from stress. The success in delivering quality patient care depends on the efficiency and motivation of the nursing personnel [13, 14]. Stress is an important part of life, and is a necessary part of coping with everyday challenges. Problems start to occur when the stress response is inappropriate to the size of the challenge. If not managed, high-stress levels result in high levels of employee dissatisfaction, illness, absenteeism, high turnover, decreased productivity, and as a result, difficulty in providing quality service to clients [15]. Stress contributes to health problems in nurses and decreases their efficiency, imposing a direct economic cost on employers and great impact on patients’ care [16].

A survey employed in Gaza-Palestine to study work-related stress among hospital nurses, using a self-administered questionnaire on population of the entire cohort of nurses who were working in the 16 hospitals in Gaza (1801 nurses; 985 males). It indicated that the most severe work-related stressors were; not enough staff to cover the unit adequately, lack of drugs and equipment required for nursing care and unpredictable staffing and scheduling respectively. The most frequent work-related stressors were; watching a patient suffers and lack of drugs and equipment required for nursing care. As sub scales, workload and death & dying were the most frequent and severe occupational stressors. Severities of occupational stressors were significantly associated with age, night shifts, specialization and qualifications. Frequencies of work-related stressors were significantly associated with hospital type, experience, specialization and night shifts [14].

A cross-sectional study on nurses’ perceived job-related stress and job satisfaction in Amman Private Hospitals through a convenience sample of 73 nurses showed the lack of enough staff to cover the unit adequately is the most stressful event perceived by the staff nurses. While by sub scale uncertainty concerning treatment was most stressful. There were no significant statistical differences in perceived job-related stress due to gender, age, and working department. However, there was significant negative relationship between the perceived job related stress and the job satisfaction of the staff nurses [17].

A study conducted in India on job stress and job satisfaction among nursing personnel through census method incorporating 210 respondents showed that experienced nurses have more stress when compared to other nurses. Stress score significantly differed across the departments, nurses working in ICU have more stress when compared to nurses working in other departments [18].

A cross-sectional study done in United Arab Emirates on a relationship between nurse’s stress and environmental – occupational factors on 216 nurses selected with random convenience sampling method among nurses working in different wards of Al-Zahra hospital showed that 42 ± 6 mean stress level. Nurses level of stress were 44.4 % had a low-stress level, 55.1 % had a moderate-stress level, and 0.5 % had a high-stress level. There were significant correlation with stress level, job satisfaction and leisure. While there were no significant correlation between stress level and age, gender, marriage status, & number of children [19].

Qualitative and quantitative survey conducted by Loo-See Beh & Leap-Han Loo to investigate the prominent causes and effects of job stress and coping mechanism among nurses in public health services in Hospital A, in the state of Selangor Darul Ehsan, in Malaysia with a total of 185 female nurses’ samples selected with simple random sampling. The results indicated that work overload, and conflict within & between groups were always a source of job stress. Centralization; low participation in decision-making was sometimes a source of stress.

While, conditions that never be sources of stress were rotating work shift and frequent relocation of unit of work [2].

A cross-sectional study on stress and its associated factors among ward nurses of Kuala Lumpur public hospital of conducted using stratified random sampling method with 114 staff nurses from 5 different departments. This study used self-administered questionnaire, the Depression, Anxiety, and Stress Scale (DASS). The prevalence of stress at the department of Medicine found higher compared to other departments studied. There was also a statistical significant relationship between the prevalence of stress and types of department. The association between prevalence of stress and age, marital status, financial status and working shift were not found to be statistically significant [10].

A cross-sectional study conducted using 150 nurses selected by purposive, non-probability sampling method to study stress and coping strategies among registered nurses working in a South African tertiary hospital revealed that registered nurses were stressed. The greatest perceived source of stress appears to be workload followed by emotional issues related to death and dying. Registered nurses seem to prefer more to positive reappraisal, planful problem solving and seeking social support [20].

One American Nurses Association study on nurses (cited on Sandra P. Thomas book entitled Transforming Nurses’ Stress and Anger) found that sexual harassment as the greater the nurse’s distress, the less likely the incident would be reported [21].

A cross sectional study conducted to explore the relationship between occupational stress and organizational commitment among nurses in selected Jordanian hospitals using systematic random sample of 150 nursing personnel reveals that there was a statistical significant occupational stress difference across working unit/department. Most of the nurses with the highest occupational stress were working in specialized units, while the least were in surgical departments. Organizational commitment is statistically significantly & negatively correlated to occupational stress [22].

The main objective of the study was to assess job-related stress and its predictors among nurses working in Jimma Zone public hospitals, South- West Ethiopia. Regarding job stress, several investigations were done in many developed countries and some African countries. However, in Ethiopia very little is known. Hence, findings from this study would help hospital administrator to weight job stress among nurses, design and implement strategies that help nurses to cope with job-stress effectively, improves the quality of life at work and their excellence in a provision of quality care. Moreover, this study will contribute its own share through providing home-based findings, especially about Jimma zone public hospital nurses. Besides, it will serve as baseline information for further research activities in the area.

## Methods

### Study area and period

The study conducted in three public hospitals found in Jimma Zone, Oromia Regional state from March 10 to April 10, 2014. Jimma is the town of Jimma zone, which is one of 18 zone of the Oromia Regional State found at 352 km from Addis Ababa, the capital city of Ethiopia, in the South western part of the country. Based on the 2007 Census conducted by the Central Statistics Agency, this Zone has a total population of 2,486,155 of these 1,250,527 are men and 1,235,628 women; with an area of 15,568.58 km^2^ [23].

In this zone there are three public hospitals namely, Jimma University specialized hospital, Shenen Gibe hospital and Limu Genet hospital. The first two are situated at Jimma town where as the later one is in Limu town, which is 72 km far from Jimma town. Except Jimma University specialized hospital both are district level. Jimma University specialized hospital plays a pivotal role in this zone and it is the only teaching and referral hospital in the southwestern part of the country, and provides specialized clinical services to about 15 million people [24]. It provides generalized service to in-patients and outpatients on a referral system in south-west part of the country.

### Study design

Institution based cross-sectional study conducted.

### Population

The Source population was all nurses who are working in Jimma Zone Public Hospitals, and the study population was all nurses who were available at work at Jimma Zone public hospitals during the data collection period.

### Inclusion and exclusion criteria

Staff nurses who were available at work at the hospital during the data collection period were included in the study. Nurses with less than 6 months of work experience excluded from the study.

### Sample size

The total population of nurse in the three public hospitals were 433 from these 73 were under six months of experience, and the remaining 360 were greater than six months of experience. Hence, the investigators conducted a census.

### Study variables

#### Independent variables

Age, sex, marital status, educational qualification, position/title, working unit, length of service, working hospital, salary, job satisfaction, and nurse-physician communication

#### Dependent variable

Job-related stress.

### Data collection instrument

Data were collected using an English version structured self-administered questionnaire developed through adaption from expanded nursing stress scale, which was developed by Gray-Toft and Anderson and Revised by Susan E. French, Rhonda Lenton, Vivienne Walters, & John Eyles in 1995, McCloskey/Mueller Satisfaction Scale (MMSS) and other relevant literatures. It contains of five parts: part-I consists of nine socio-demographic questions. Part-II contains 26 items that help to measure job-related stress among nurses. The items are divided into seven major sub scales. Thus are: workload (has five items), lack of support (has three items), conflict (has four items), uncertainty regarding patient treatment (has four items), dealing with death & dying (has three items), inadequate preparation (has three items), organizational decisions (has three items) and sexual harassment (has one item). A four-point Likert item represents each of the items. Likert item has options from 1to 4 where 1 represents “if the condition is never a cause of stressful”, 2 “if the condition is sometimes stressful”, 3 “if the condition is frequently stressful” and 4 “if the condition is always stressful”. Respondents asked to indicate how often the causes of job stress stated in the questionnaire occurred in their work life. The higher the score, the more the respondent agrees that the situation is stressful. The total stress score that provides the overall levels of stress among nurses obtained by adding all the scores on 26 items together. Part III & IV are regarding nurse-physician communication related factors; Part III- regarding personal and organizational factors has nine items and Part IV: measures mutual understanding at work has three items. Part V: Job satisfaction measured through the McCloskey/Mueller Satisfaction Scale (MMSS) that has 29 questions. This study questionnaire was pretested with five percent of sample size on nurses working at Woliso St. Luke Hospital before the start of actual data collection and necessary comments and feedback taken and incorporated. Pretest overall Cronbach’s alpha score for overall stress measuring items 0.84, nurse-physician-related factors (Organizational factors 0.88, Personal factors 0.92, & Mutual understanding 0.78) and job satisfaction had 0.90.

### Data collection personnel

Five diploma nurses working outside the study hospitals recruited and assigned: three for Jimma University Specialized Hospital, one for Shenen Gibe Hospital and one for Limu Genet Hospital. Roles of data collectors were distributing questionnaires for nurses in their respective working unit and shifts (morning, late afternoon and night shifts) and collecting questionnaires from head nurses’ office. Subjects return questionnaires after completion to head nurses’ office. Data collectors trained and oriented for one day on the questionnaire and the way of data collection.

### Data quality control

The collected data reviewed and checked for completeness by the facilitator/data collectors and principal investigators. For each shift, the questionnaire distributed after the purpose of the study explained and told to return when they finished. To assure anonymity, code numbers given on completed questionnaires after they return to the investigator.

### Data processing and analysis

The data were edited, entered into Epi-Data version 3.1 and exported to SPSS Version 20 for analysis. The results were summarized and presented by tables, and charts. Percentage, frequency and mean calculated. For each sub scale, the participant’s responses on each item score summed: a stress score ranging from a minimum of 26 and maximum score of 104. The higher the sum the more the stressed the nurse. Percentages mean score of job-related stress for sub scales calculated by using the following formula:

$$ \mathrm{Percentage}\ \mathrm{mean}\ \mathrm{score}\ \mathrm{of}\ \mathrm{job}\hbox{-} \mathrm{related}\ \mathrm{stress}\kern1em =\kern0.5em \left\{\kern0.5em \frac{\mathrm{Actualcomputed}\ \mathrm{mean}\ \mathrm{score}}{\mathrm{Maximum}\ \mathrm{potential}\ \mathrm{score}}\right\}\kern0.5em \mathrm{x}\kern0.5em 100 $$

Bivariate and multivariable linear regressions were done to see the association between the predictor and the outcome variables. ß-coefficients used to show independent predictors of stress. *P*-value less than 0.05 taken as statistically significant.

## Results

A total of 341 nurses who are working in three Jimma Zone public hospitals (Jimma University specialized hospital, Shenen-Gibe & Limu Genet Hospital) were given to respond to the self-administered questionnaire. The response rate was 92.38 % (315) nurses responded to the given questionnaire. Respondents were 85.7 % (270) from Jimma University specialized hospital, 6.3 % (20) from Shenen-Gibe and 7.9 % (25) from Limu Genet hospital. Minimum age of the respondents were 21 years, mean age 27.95 ± 6.83 years and a maximum of 58 years old, majority of respondents were married 43.2 % (136) (Table 1).

**Table 1:** The socio-demographic characteristics among nurses working in Jimma Zone Public Hospitals, South-west Ethiopia, 2014

*N* = 315			
		Number	Percent
Hospital	Jimma University Specialized Hospital	270	85.7
	Shenen Gibe Hospital	20	6.3
	Limu Genet Hospital	25	7.9
Gender	Male	160	50.8
	Female	155	49.2
Marital status	Married	136	43.2
	Single	172	54.6
	Divorced	7	2.2
Working Unit/department	Medical ward	54	17.1
	Surgical ward	65	20.6
	Intensive Care Unit (ICU)	10	3.2
	Major Operation room	21	6.7
	Psychiatry	9	2.9
	Pediatrics	45	14.3
	Obstetrics & Gynecology	23	7.3
	Ophthalmology	13	4.1
	Chronic Illness Follow Up Clinic	19	6.0
	OPD	56	17.8
Age	≤24 years	103	32.7
	25-29 years	151	47.9
	30-34 years	18	5.7
	≥35 years	43	13.7
Work Experience in Nursing	$\frac{\mathbf{1}}{\mathbf{2}}\mathbf{year}$ - 5 years	243	77.1
	6–10 years	38	12.1
	>10 years	34	10.8
Rank/position	Staff nurse	283	89.8
	Head nurse	27	8.6
	Supervisor nurse	2	0.6
	Matron Nurse	3	1.0
Educational Qualification	Diploma	194	61.6
	BSc.N	121	38.4
Salary	≤1427 Birr	119	37.8
	1428–1800 Birr	45	14.3
	1801–2250 Birr	129	41.0
	≥2251 Birr	22	7.0

The overall level of job related stress has been calculated by adding the 26 items scores. A minimum score of 26 and a maximum score of 104 was possible, the higher the score indicates the higher level of stress. The minimum total stress score was 28 and maximum of 99 (Table 2).

**Table 2:** Descriptive statistics for overall job related stress score among Nurses working in Jimma Zone Public Hospitals, South-west Ethiopia, 2014

(*n* = 315)	Minimum	Maximum	Mean	SD	Percentage Mean
Overall Job related stress score	28	99	58.46	12.62	56.28

**Fig. 1:**
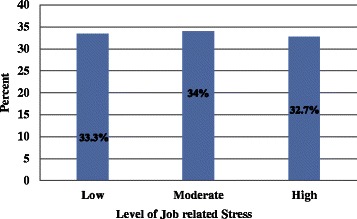
Bar graph showing the level of job related stress among Nurses working in Jimma Zone Public Hospitals, South-west Ethiopia, 2014

Based on a data driven tertial rank classification a lowest score indicates a low stress (stress score 28–52), a medium score indicates a moderate stress (stress score 53–63) & a high score indicates a high stress (stress score 54–99); 33.4 % (105) of nurses had low stress, 34 % (107) of nurses had moderate stress, and 32.7 % (103) of nurses had high stress (Fig. 1).

Based on mean score of each item of job-related stress the most stressful conditions rated among nurses in descending order were: the death of a patient (2.87 ± 1.04), watching a patient suffer (2.61 ± 1.05), centralization; low participation in decision making (2.51 ± 0.99), a physician not being present in a medical emergency (2.44 ± 1.01), and not enough staff to cover the unit adequately (2.43 ± 0.95).

The least stressful conditions rated among nurses in descending order were: being sexually harassed/requests for sexual favors, and other verbal or physical conduct of a sexual nature in work environment (1.85 ± 1.03), difficulty in working with a specific nurse in the unit (1.9 ± 0.94), not enough time to respond to the needs of patients’ families (2.06 ± 0.86), feeling as my support is helpless in the case of a patient who fails to improve (2.08 ± 1), and frequent change of working unit (2.09 ± 0.91) (Table 3).

**Table 3:** Frequency and percentage distribution of each item of Job related stress among Nurses working in Jimma Zone Public Hospitals, South-west Ethiopia, 2014 (*N* = 315)

Sub scale	Item	Never stressful		Sometimes stressful		Frequently stressful		Always/very frequently/stressful		Mean	Std. Dev
		n	%	n	%	n	%	n	%		
Work load	Not enough staff to adequately cover the unit.	39	12.4	113	35.9	106	33.7	57	18.1	2.57	0.926
	Not enough time to complete all of my nursing tasks.	84	26.7	125	39.7	81	25.7	25	7.9	2.15	0.906
	Not enough time to provide emotional support to the patient.	67	21.3	141	44.8	82	26.0	25	7.9	2.21	0.866
	Too many non-nursing tasks required, such as clerical work.	48	15.2	122	38.7	97	30.8	48	15.2	2.46	0.928
	Not enough time to respond to the needs of patients’ families.	79	25.1	146	46.3	60	19.0	30	9.5	2.13	0.899
Conflict	Conflict with physician.	83	26.3	142	45.1	54	17.1	36	11.4	2.14	0.94
	Disagreement concerning the treatment of a patient.	85	27.0	144	45.7	59	18.7	27	8.6	2.09	0.89
	Conflict with a nurse supervisor.	103	32.7	111	35.2	54	17.1	47	14.9	2.14	1.04
	Difficulty in working with a specific nurse in the unit.	132	41.9	108	34.3	50	15.9	25	7.9	1.90	0.94
Lack of support	Lack of opportunity to talk openly with other unit personnel about problems in the unit.	70	22.2	101	32.1	88	27.9	56	17.8	2.41	1.023
	Lack of opportunity to share experiences and feelings with other personnel in the unit.	86	27.3	123	39.0	68	21.6	38	12.1	2.18	0.97
	Lack of support of my immediate supervisor.	110	34.9	103	32.7	56	17.8	46	14.6	2.12	1.04
Uncertainty regarding patient treatment	Inadequate information from a physician regarding the medical condition of a patient.	68	21.6	121	38.4	86	27.3	40	12.7	2.31	0.95
	A physician ordering what appears to be inappropriate treatment for a patient.	63	20.0	121	38.4	86	27.3	45	14.3	2.36	0.96
	A physician not being present in a medical emergency.	63	20.0	107	34.0	88	27.9	57	18.1	2.44	1.01
	Not knowing what a patient or a patient’s family ought to be told about the patient’s condition & treatment.	75	23.8	149	47.3	68	21.6	23	7.3	2.12	0.85
Dealing with death & dying	Feeling as my support is helpless in the case of a patient who fails to improve.	110	34.9	109	34.6	58	18.4	38	12.1	2.08	1.00
	Watching a patient suffer.	52	16.5	106	33.7	71	22.5	86	27.3	2.61	1.05
	The death of a patient.	30	9.5	103	32.7	60	19.0	122	38.7	2.87	1.04
Organizational decisions	Frequent change of unit of work.	87	27.6	142	45.1	56	17.8	30	9.5	2.09	0.91
	Rotating work shift.	94	29.8	125	39.7	51	16.2	45	14.3	2.15	1.00
	Centralization; low participation in decision making.	53	16.8	111	35.2	88	27.9	63	20.0	2.51	0.99
Inadequate preparation	Feeling inadequately prepared to help with emotional needs of a patient.	69	21.9	145	46.0	70	22.2	31	9.8	2.20	0.89
	Being asked a question by a patient for which I do not have satisfactory answer.	81	25.7	134	42.5	72	22.9	28	8.9	2.15	0.90
	Feeling inadequately prepared to help with the emotional needs of a patient’s family.	66	21.0	141	44.8	82	26.0	26	8.3	2.22	0.86
Sexual harassment	Being sexually harassed/requests for sexual favors, and other verbal.	163	51.7	69	21.9	51	16.2	32	10.2	1.85	1.03

Out of the eight sub scales, the highest level of job-related stress was on the sub scale of dealing with death & dying with a mean score of 62.94 % followed by uncertainty regarding patient treatment 57.72 % and work overload 57.6 %. While job-related stress from sexual harassment had the lowest mean score of 46.19 % (Table 4).

**Table 4:** Percentage mean score of Job related stress sub scale among Nurses working in Jimma Zone Public Hospitals, South-west Ethiopia, 2014 (*n* = 315)

Sub scale	Mean subscale score	Std. Dev	Mean %(%)	Minimum (%)	Maximum (%)
Stress from dealing with death & dying	7.55	2.407	62.94	25	100
Stress from uncertainty regarding pt. treatment	9.23	2.694	57.72	25	100
Stress from workload	11.52	2.876	57.6	25	100
Stress from organizational decisions	6.75	1.989	56.27	25	100
Stress from lack of support	6.72	2.273	55.98	25	100
Stress from inadequate preparation	6.57	2.073	54.71	25	100
Stress from conflict	8.27	2.742	51.67	25	100
Stress from sexual harassments	1.85	1.032	46.19	25	100
Stress from sexual harassments	1.85	1.032	46.19	25	100
Stress from conflict	8.27	2.742	51.67	25	100
Stress from inadequate preparation	6.57	2.073	54.71	25	100
Stress from lack of support	6.72	2.273	55.98	25	100
Stress from organizational decisions	6.75	1.989	56.27	25	100
Stress from workload	11.52	2.876	57.60	25	100
Stress from uncertainty regarding pt. treatment	9.23	2.694	57.72	25	100
Stress from dealing with death & dying	7.55	2.407	62.94	25	100
Overall Percentage mean score of job related stress	58.46	12.62	56.28		

A one-way ANOVA showed statistically significant mean difference of overall job related stress across working unit. F (9, 305) = 2.2450, p = 0.010 (Table 5).

**Table 5:** Descriptive statistics for respondents overall job related stress score with in working unit/department

Working unit	Number	Mean	Std. Deviation	Between groups F	*p*
Medical ward	54	59.33	10.944	2.450	0.010*
Surgical ward	65	55.03	10.605		
Intensive Care Unit (ICU)	10	60.80	9.390		
Major Operation room	21	64.05	13.728		
Psychiatry	9	59.44	12.187		
Pediatrics	45	59.22	15.325		
Obstetrics & Gynecology	23	59.65	14.850		
Ophthalmology	13	53.92	12.919		
Chronic Illness Follow Up Clinic	19	51.05	8.316		
OPD	56	61.38	12.703		
Total	315	58.46	12.616		

*p-value < 0.05

Regarding job satisfaction the minimum score was 29 and the maximum score was 116 while the overall mean of job satisfaction of the study participating nurses in Jimma zone public hospitals in this study was (67.43 ± 13.85) with a mean score of 58.13 %.

The following variables were entered in to bivariate linear regression analysis independently; working hospital, sex, age, marital status, length of service, educational qualification, position, salary, working unit (medical ward, surgical ward, intensive care unit, major operation room, psychiatry, pediatrics ward, obstetrics & gynecology, ophthalmology, chronic illness follow-up clinic, outpatient department), mutual understanding at work, job satisfaction. From these those predictor variables that had *p*- value < 0.25 taken as candidate variables for multiple linear regression models (Table 6).

**Table 6:** Multiple linear regression predicting Job related stress among nurses working in Jimma Zone Public Hospitals, South-west Ethiopia, 2014

Predictor variables	Unstandardized coefficients		T	*p*-value	95 % confidence interval for β	
	β	Std. Error			Lower Bound	Upper Bound
(Constant)	74.416	4.975	14.959	0.000	64.627	84.204
Sex	0.231	1.269	0.182	0.856	−2.267	2.729
Age	0.086	0.094	0.916	0.361	−0.099	0.271
Mutual understanding at work	−0.497	0.221	−2.25	0.025*	−0.932	−0.062
Job satisfaction	−0.343	0.046	−7.435	0.000**	−0.434	−0.252
Working unit/department						
Chronic illness follow up clinic	−5.964	2.684	−2.222	0.027*	−11.245	−0.682
Outpatient department	3.309	1.677	1.973	0.049*	0.009	6.609

Dependent Variable: Overall Stress score

Max VIF = 1.092, *p < 0.05, **p < 0.001, Adjusted R^2^ = 0.243, *F* = 15.415, *p* = 0.000

Enter method was used and step by step predictor variable with the largest *p*-value removed until the final model built. The overall model was significant (*F = 15.415, p = 0.000)* and explains 24.3 % of the variance in the outcome variable (Job related stress). As shown on Table 6, the multiple variable linear regression model indicated that mutual understanding at work between nurse & physician, job satisfaction and working in a chronic illness follow up clinic had inverse association with overall job-related stress which is statistically significant, i.e. *p* = 0.025, *p* = 0.000, & *p* = 0.027 respectively. Working in an outpatient department had a positive & statistically significant association with job-related stress.

In this study working hospital, sex, age, marital status, length of service, educational qualification, position, salary, and working unit (medical ward, surgical ward, intensive care unit, major operation room, psychiatry, pediatrics ward, obstetrics & gynecology, ophthalmology) were not found to be statistically significant predictors of job related stress. A unit increase on mutual understanding at work between nurse and physician would likely decrease job-related stress by −0.497. A unit increase on job satisfaction would likely decrease 0.343 on job-related stress. Working in chronic illness follow up clinic would likely decrease 5.964 on job-related stress than other units. While working in the outpatient department would likely increase 3.309 on job-related stress than other units.

Comparing the contribution of each independent variable, the largest beta coefficient belongs to working in the chronic illness follow up clinic (ß = 5.964, *p* = 0.027). This variable makes the strongest unique contribution in explaining the dependent variable (job-related stress), when the variance explained by all other variables in the model is controlled for. The ß value for job satisfaction was the lowest (ß = 0.343, *p* = 0.000), indicating that it made less of a unique contribution to the model when the variance explained by all other variables in the model is controlled for.

## Discussion

This study respondents (*n* = 315) indicated mean overall job-related stress level of 58.08 ± 12.62. Moreover, 33.4 % of nurses had low stress, 34 % moderate stress and 32.7 % had high stress. In contradiction to current study, a study done in United Arab Emirates showed that mean stress level 42 ± 6 & nurses level of stress were 44.4 % had a low stress level, 55.1 % had a moderate stress level, and 0.5 % had a high stress level [19] which shows the overall mean stress score & high level stress lower than this study. This discrepancy may be due to a staffing/workload difference of these two studies.

The highest stressful condition that nurses rated as always stressful were the death of a patient followed by watching a patient suffer. This may be due to linking death with clinical failure. Not enough staff to cover the unit adequately was frequently stressful condition. In line with this, a study done in Jordan showed that the lack of enough staff to adequately cover the unit is the most stressful event perceived by the staff nurses [17].

The least stressful conditions rated among nurses were being sexually harassed in work environment. In contradiction to this American Nurse Association study found that sexual harassment as the greater the nurse’s distress, the less likely the incident would be reported [21] this difference may be due to the sensitive nature of the subject matter inquired.

This study indicated that higher overall job related stress from dealing with death & dying sub scale followed by uncertainty regarding patient treatment and workload which is consistent with a study done in Gaza-Palestine that stated death & dying and workload as the most frequent and severe occupational stressors [14]. In line with this, a study conducted in the South African tertiary hospital revealed that the greatest perceived source of stress appears to be workload followed by emotional issues related to death and dying [20].

This study indicated that there is no variation in overall job-related stress across age & sexes. This finding is consistent with a study done in Kuala Lumpur that reveals that age was not found to be statistically significant [10]. Furthermore, supported by a study done in Jordan showed that there were no significant statistical differences in perceived job-related stress due to gender [17].

Finding of this study also indicated that there is no variation in job-related stress due to educational qualification, position, working hospital, & length of service (experience in nursing). Contradictory to this finding, on length of service a study conducted in India indicated that nurses with a total nursing experience of 11–20 years feel more stress [18] this could be due to age distribution of this study mean age 27.95 ± 6.83 years which indicates study subjects are younger.

Current study showed that job related stress variation between working units/departments: nurses working in major operation room, outpatient department, obstetrics & gynecology, pediatrics & medical ward had higher stress in the order stated here than those nurses who are working in chronic illness follow up clinics. However, this finding is inconsistent with Jordan’s study which reveals that there were no significant statistical differences in perceived job-related stress due to work unit [17]. This discrepancy may be due to man-power/staff allocation differences across units.

Nurses working at outpatient department had higher stress than those in a chronic illness follow up clinics from organizational decisions this would be due to random and frequent rotation of nurses within outpatient department units in short intervals.

In general, current study showed that there existed overall job related variation between working units/departments. In line with the present study findings from Jordan’s study supports that occupational stress difference across working unit [22].

In this study; working unit/department particularly working in the chronic illness follow-up clinic & outpatient department, mutual understanding at work between nurse & physician, and job satisfaction were predictor variables for overall job related stress. However, age & sex were not predictors. As revealed in this study, working in the chronic illness follow-up clinic had significantly lower job related stress than other units. This may be due to nurse who are working in this unit help patients who are stable & ambulatory patient who need follow-up and this would not make nurses stressed when compared to units where there are critical care, dealing with death and dying etc. While to the contrary working in outpatient department found to be more stressful than other units this may be due to presence of emergency departments like medical emergency & surgical emergency outpatient departments where there would be management of casualty, unexpected numbers of patients at any time than other units and this would possibly make them more stressful. This also supported by a study done in emergency department Nurses in Taiwan which reveals stresses are inevitable in all emergency departments [25].

Current study showed that the presence of mutual understanding between nurse & physician decreases job related stress among nurses. This could be because of the relationship between mutual understanding and collaboration where true collaboration requires mutual understanding, open & honest communication, and equitable, shared decision making powers [26] which in turn these would decrease job related stress among nurses by increasing nurses’ autonomy & decreasing conflict between them. Besides, the presence of collaborative practice also improves job satisfaction [27, 28].

In this study job satisfaction was also a predictor of job related stress in which their association is inverse; as job satisfaction increases nurse’s stress would decrease. This finding is supported by a study done in Sao Paulo that reveals dissatisfaction with work could lead to stress [29]. Furthermore, similar to a study done in Kampala, Uganda that reveals there were significant negative relationships between occupational stress job satisfaction [30].

This study limitation is that the generalization of the findings is limited to nurses working in public hospitals. Hence, it is not generalizable for nurses who are working in health center & private clinics.

## Conclusion

This study indicated mean overall level of job-related stress among nurses working in Jimma Zone public hospital was high among one-third. Job-related stress from dealing with death & dying; uncertainty regarding patient treatment and workload were higher in descending order while from sexual harassment & conflict least.

Overall job- related stress varies across working unit. Besides, it was higher among nurses who are working in the outpatient department & least among nurses working in chronic illness follow up clinics. Not enough staff to cover the unit adequately was frequently stressful condition.

Working in chronic illness follow-up clinic, mutual understanding at work between nurse & physician and job satisfaction were negatively associated predictors of job-related stress.

## Abbreviations

BSc.N, bachelor of science in nursing; ICU, intensive care unit; OPD, outpatient department; SPSS, statistical package for the social science
